# 

**Published:** 1893-08

**Authors:** 


					﻿THE
—  	\S. G. O.
Southern Medical Record
A Monthly Journal of Medicine and Surgery.
VOL. XXIII.
Atlanta, Ga., August, 1893.
NO 8>
EDITORS:
A. W. GRIGGS, M. D.
3. mcfadden gaston, m. d.
WILLIS F. WESTMORELAND, M. D.
LOUIS H. JONES, Mi D.
DAN. H. HOWELL, M. D., Bus. Mgr.
Entered at the Postoffice at Atlanta, Ga., as Second-Class Matter.
Mutual Printing Company, Publishers, 27 East Hunter St., Atlanta, Ga.
				

